# Interventions to mitigate pain and reduce skin impedance during neuromuscular electrical stimulation

**DOI:** 10.1016/j.cnp.2026.02.005

**Published:** 2026-02-13

**Authors:** Philip Wackendal, Ines Shopi, Robin Juthberg, Paul W Ackermann

**Affiliations:** a Integrative Orthopaedic Laboratory, Department of Molecular Medicine and Surgery, Karolinska Institutet, Stockholm, Sweden; b Department of Trauma, Acute Surgery and Orthopaedics, Karolinska University Hospital, Stockholm, Sweden

**Keywords:** Neuromuscular electrical stimulation, Skin–electrode interface, Pain perception, Exfoliation, Hydrogel electrodes, Venous thromboembolism, Gel pad electrodes

## Abstract

**Objective:**

High skin–electrode impedance limits neuromuscular electrical stimulation (NMES) by increasing discomfort and impairing efficacy. This study compared the effects of mechanical exfoliation and gel pad (hydrogel) application on skin impedance, NMES current intensity, and pain perception.

**Methods:**

In a within-subject design, 49 healthy participants (28 males, 21 females) received NMES on both calves with randomized interventions (dry electrode, gel pad, exfoliation, combined). Impedance, NMES current intensity and pain (Numerical Pain Rating Scale, NPRS, 0–10 (no-maximum pain)) at plantar flexion (PF) were recorded.

**Results:**

Gel pads produced the largest impedance reduction (∼10 kΩ) in participants reaching PF at lower intensities (PF range: 5–20 mA). All interventions significantly reduced impedance (∼1.5 kΩ) and current intensity (∼1 mA) at PF versus dry electrodes (p < 0.001). Gel pads, alone or combined with exfoliation, reduced pain by ∼ 1 NPRS point (p < 0.001), while exfoliation alone had no effect. Over 20% of participants reported persistent pain (NPRS ≥ 4) despite interventions. Higher impedance was significantly associated with lower physical activity level.

**Conclusion:**

Gel pads effectively reduced both impedance and pain during NMES, while exfoliation reduced impedance without affecting pain.

**Significance:**

Hydrogel pads improve NMES tolerability, yet persistent discomfort warrants improved skin–electrode interfaces.

## Highlights

• Gel pads lower skin impedance and improve comfort during neuromuscular stimulation.
• Skin exfoliation lowers impedance but provides no benefits to comfort.
• Over 20% report moderate-to-high pain (≥4/10 scale) despite gel pad intervention.

## 1 Introduction

Neuromuscular electrical stimulation (NMES) is widely used in rehabilitation and sports medicine to enhance muscle strength, prevent atrophy, and support functional recovery (Doucet et al., 2012, Jones et al., 2016, O’Connor et al., 2018). More recently, NMES applied to the calf has been explored as a prophylactic intervention for deep vein thrombosis (DVT), primarily by enhancing venous return (Hajibandeh et al., n.d.; Praxitelous et al., 2018, Senin-Camargo et al., 2022, Williams et al., 2016). Despite its broad clinical utility, the efficacy and tolerability of NMES vary considerably among individuals (Maffiuletti et al., 2018, Praxitelous et al., 2018). A key technical barrier to consistent and comfortable stimulation is high skin impedance, which can impair current delivery, reduce stimulation efficiency, and increase discomfort or pain during treatment (Zhou et al., 2015).

To address these challenges, several strategies have been proposed to reduce impedance at the skin-electrode interface. Hydration techniques and conductive gels are commonly used to improve contact quality, while mechanical preparation of the skin, such as exfoliation, may further lower impedance by removing the outermost layer of dead cells and surface oils (Jafari et al., 2025). However, the comparative effectiveness of these approaches remains unclear, particularly under controlled environmental and stimulation conditions. Moreover, while some interventions reduce impedance or current thresholds, their influence on perceived pain has been inconsistently reported (Euler et al., 2021, Euler et al., 2022).

Known predictors of higher skin impedance include dry skin, a thick stratum corneum (Murphy et al., 2021), and low sudomotor activity (Bîrlea et al., 2014). Recent findings also suggest that training status influences NMES responsiveness, as higher physical activity has been associated with a greater number of motor points on the muscle (Flodin et al., 2025). Whether physically active individuals also exhibit lower skin impedance has not yet been investigated.

The primary aim of this study was to systematically evaluate the effects of mechanical exfoliation, hydrogel (gel pad) application, and their combination, relative to a dry electrode condition on skin impedance, NMES current intensity, and pain perception during stimulation of the calf musculature. A secondary objective was to identify individual-level predictors of impedance reduction, including potential indicators of suboptimal NMES response. Such predictors may help to distinguish individuals less likely to tolerate or benefit from NMES, and support simple screening strategies to guide personalized NMES use.

## 2 Material and methods

### 2.1 Study design

**Fig. 1:**
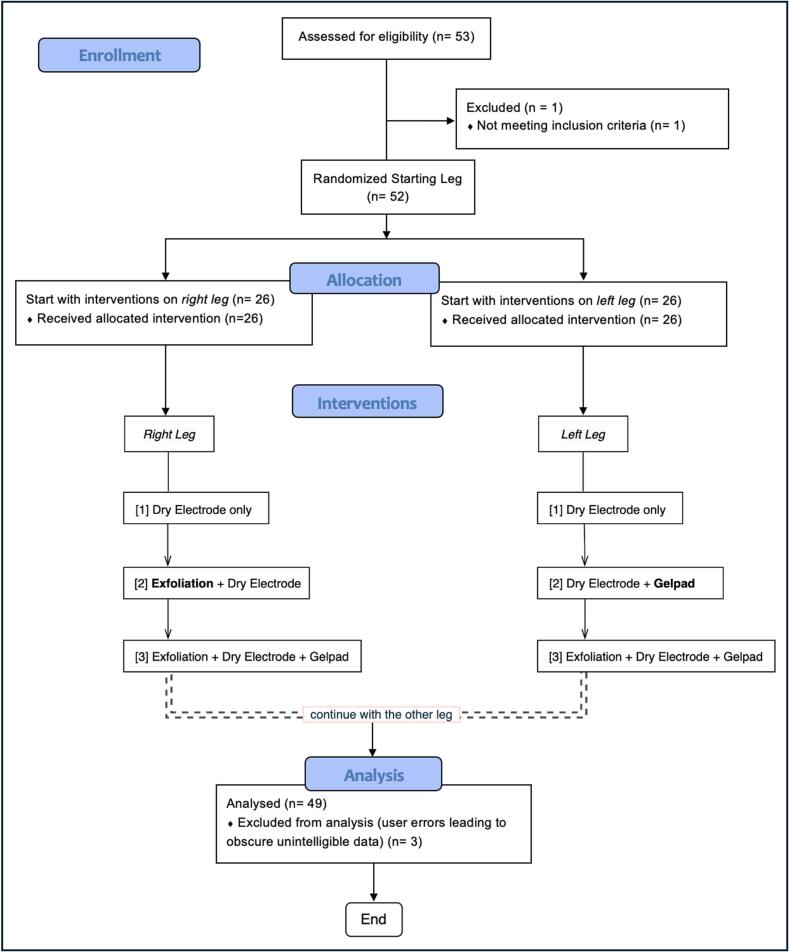
CONSORT flow diagram illustrating participant progression through NMES interventions applied to the left and right legs.

This controlled, within-subject experimental study was conducted from September 1 to December 31, 2024, at the Department of Trauma and Acute Orthopedic Surgery, Karolinska University Hospital, Solna. All testing was carried out in a standard hospital wardroom with controlled lighting, temperature (22–24 °C), and humidity (40–60%) to minimize environmental effects on impedance measurements. Electrode placement was standardized using anatomical landmarks, and skin preparation followed a uniform protocol. The starting leg was randomized, and each participant underwent all intervention conditions in a counterbalanced design, with one sequence applied to the right leg and a different sequence to the left leg, enabling within-subject and cross-leg comparisons (Fig. 1).

### Ethical approval

This study was conducted in accordance with the Declaration of Helsinki and approved by the Institutional Review Board (Dnr: 2024–06543-02). All participants provided written informed consent after receiving both verbal and written information regarding the study’s purpose, procedures, potential risks, and their right to withdraw at any time without consequences.

### 2.2 Study participants

A total of 53 healthy volunteers (29 females, 24 males), aged 22–63 years, were recruited via institutional postings and online advertisements. Participants had no history of neuromuscular disorders, skin conditions, or contraindications to NMES. Exclusion criteria included skin lesions, chronic skin-affecting illnesses, recent use of topical medications, and contraindications to electrical stimulation (e.g., pacemakers, epilepsy, or cardiovascular disease). One participant was excluded due to abrasions. Two participants discontinued during specific interventions (dry electrode or combined gel pad and exfoliation on the left leg) but completed the remaining tests. Three additional datasets were excluded due to measurement errors. Final analysis included data from 49 participants.

#### 2.2.1 Physical activity level

Participants self-reported their physical activity levels using the Frändin/Grimby physical activity scale, which categorizes activity into six grades:

Grade 1 = No physical activity.

Grade 2 = Mostly sedentary, sometimes shorter walks.

Grade 3 = Light physical activity 2 to 4 h per week.

Grade 4 = Exercise 1 to 2 h per week.

Grade 5 = Exercise 3 or more hours per week.Grade 6 = Trains hard several times per week.

This scale provided a standardized measure to quantify and compare habitual physical activity among participants.

#### 2.2.2 US navy body fat method

Body fat percentage was estimated using the US Navy Body Fat Method, which incorporates height (self-reported), neck circumference, waist circumference and hip circumference (for women only). This method is widely recognized for its reliability in estimating body fat.

### 2.3 Testing protocol

The tests were performed by three trained examiners. Participants were positioned prone with extended legs on a gurney. Precut conductive silicone electrodes (FIAB PG971/40G (4 × 4 cm), Italy) were placed over the gastrocnemius muscle of both legs. The electrodes were stored in a controlled environment to maintain their adhesive and conductive properties.

#### 2.3.1 Electrode placement

**Fig. 2:**
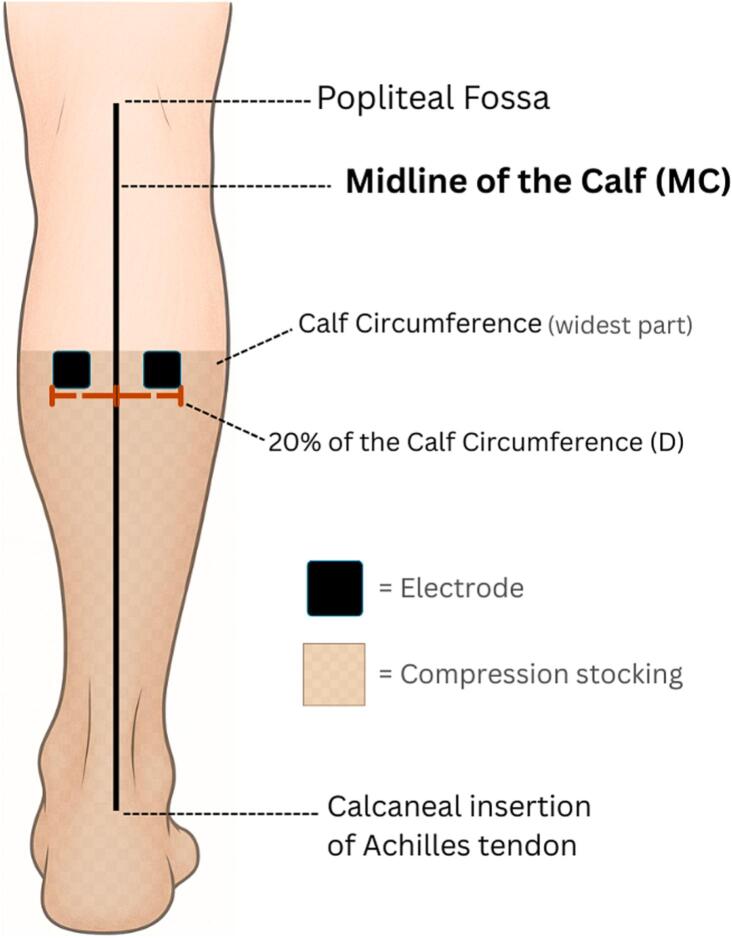
A. Schematic figure of the electrode setup along with the anatomical landmarks used to ensure consistent placements throughout the study.

The examiner identified the location of the largest circumference of the calf through tape measuring and demarcated it with a horizontal line using a skin-safe pen. The circumference was measured to the nearest millimeter. The length of the calf was measured from the midline of fossa poplitea to midline of the calcaneal insertion of the Achilles tendon, and a line was drawn to define the midline of the calf (MC). To determine the electrode placement in relation to the MC, a distance (D), was calculated as 20% of the calf circumference at the widest part (e.g., D = 0.2 × max circumference). A mark was made D cm lateral and medial to the MC where the MC crosses the line that marks the widest part of the calf. The midline of the electrode is placed on the line that marks the widest part of the calf, and the medial border of the electrode was placed D cm from the MC. The outlines of both electrodes were then marked, enabling consistency of electrode placements between participants and interventions. Equal compression was applied on each test using Class I-graduated medical compression stockings (15–21 mmHg), sized appropriately for each participant’s calf circumference (Mabs COTTON KNEE, Karo Pharma AB, Sweden) (Fig. 2).

#### 2.3.2 Skin exfoliation

Skin preparation was only initialized at the exfoliation step of the procedure. The skin was prepared using a standardized exfoliation protocol to reduce impedance and ensure consistent electrode–skin contact. An exfoliating glove (Apoliva Exfoliation Glove, Apoteket AB, Sweden) was used to exfoliate the target area with moderate pressure in a back-and-forth motion. The exfoliation continued until one of the following criteria was met: (1) the participant reported a pain rating of 4 on the Numerical Pain Rating Scale (NPRS), indicating moderate pain, or (2) the exfoliation area exhibited visible erythema (redness), indicating increased blood flow and potential removal of the stratum corneum.

#### 2.3.3 Interventions

Each participant underwent three sequential electrode interventions on the calf of each leg. The starting leg was randomized, and both legs were ultimately tested. While the sequence of interventions differed between the right and left leg, it remained consistent among all participants.

#### 2.3.4 Intervention types

• **Dry Electrodes**: Conductive silicone electrodes were applied directly to the skin without prior preparation.
• **Gel Pad Electrodes**: A precut gel pad (FIAB PG271/40 (4x4 cm), Italy) was attached to the conductive silicone electrode and the skin.
• **Skin Exfoliation**: The skin was exfoliated as described above, followed by the placement of a conductive silicone electrode alone or with the combination of a conductive silicone electrode and a gel pad depending on the leg tested.

**The Right Leg** had the following test sequence:

1. *Dry electrode alone.*
2. *Skin exfoliation followed by re-application of a conductive silicone electrode.*
3. *Gel pad attached to the conductive silicone electrode on previously exfoliated skin.*

**The Left Leg** had the following test sequence:

1. *Dry electrode alone.*
2. *Gel pad attached to the conductive silicone electrode.*
3. *Skin exfoliation followed by re-application of a gel pad and conductive silicone electrode.*

### 2.4 NMES pulse delivery

NMES was delivered using an Enraf Nonius Endomed 484 NMES device (Enraf Nonius, Rotterdam, Netherlands). A biphasic, square-form pulse was used with the following parameters: frequency of 36 Hz, phase duration of 350 μs (total pulse duration: 700 μs, ramp-up time 0 s, ON time 1 s, ramp-down time 0 s, and OFF time 5 s, according to previously recommended protocols in the literature (Ackermann et al., 2024).

Stimulation intensity was gradually increased in 2.5 mA increments until a plantar flexion of the foot was visually confirmed by the examiner. Once a plantar flexion was confirmed, the stimulation intensity was reduced in 1 mA increments until plantar flexion was no longer visible. The lowest NMES intensity that still elicited a visible plantar flexion was recorded as the NMES current required for plantar flexion (IaPF).

#### 2.4.1 Voltage and current measurements

Voltage (V) and current (mA) readings were recorded simultaneously using a Tektronix TDS3014B oscilloscope (Tektronix, Beaverton, OR, USA). A Tektronix P52XXA Series P5200A Oscilloscope Probe was used for voltage measurements, and a Tektronix TCP202 DC-coupled current probe was used for current measurements. Skin impedance (kΩ) was calculated using Ohm’s law (Z = V/I) and averaged across three trials per intervention at each NMES level. The impedance recorded at the lowest NMES intensity that elicited a visible plantar flexion was designated as ZaPF. The oscilloscope was calibrated prior to each testing session, and any deviations or anomalies were corrected before proceeding with data collection.

#### 2.4.2 Numerical Pain Rating Scale (NPRS)

Discomfort or pain perception was assessed using the Numerical Pain Rating Scale (NPRS), a whole number scale ranging from 0 (no pain) to 10 (maximum pain). The NPRS score recorded at the lowest NMES intensity that produced a visible plantar flexion was designated as PaPF. Participants were provided with standardized instructions on how to use the NPRS and were familiarized with the scale prior to testing.

#### 2.4.3 NMES intensity threshold and safety monitoring

The minimum NMES intensity (mA) required to elicit plantar flexion was recorded for each intervention. Each measurement was repeated three times, and the values were averaged for analysis. Once plantar flexion was achieved, testing for the current condition (e.g., with dry electrodes) was terminated, and the subsequent intervention was initiated.

Safety precautions included continuous monitoring of participants for adverse effects such as skin irritation or muscle fatigue. Participants were instructed to report any discomfort immediately and were informed that they could withdraw from the study or discontinue any specific test at any time without providing a reason. No adverse reactions or participant withdrawals occurred during the study.

### 2.5 Statistical analysis

All statistical analyses were performed using SPSS version 29 (IBM Corp., Armonk, NY, USA) and R version 4.4.2. Descriptive statistics are presented as mean ± standard deviation (SD) for normally distributed variables, and median (interquartile range, IQR) for non-normally distributed variables. Normality of continuous outcomes was assessed using the Shapiro–Wilk test.

Because the paired differences in the following variables: ZaPF and IaPF, did not meet the normality assumption for any of the interventions, within-subject comparisons across interventions were conducted using the non-parametric Wilcoxon signed-rank test. Changes in PaPF were evaluated using the sign test, as NPRS data are ordinal and the paired differences did not satisfy the symmetry assumption required for the Wilcoxon signed-rank test.

Exploratory multiple linear regression models with stepwise selection were employed to examine associations between ZaPF and potential predictors (age, sex, body composition, physical activity level, applied NMES current, and starting leg). Model fit was reported as R^2^ and adjusted R^2^.

A two-sided p-value < 0.05 was considered statistically significant.

## 3 Results

### 3.1 Participants

A total of 49 participants (28 males, 21 females; mean age: 37.4 ± 12 years) were included in the analysis. The mean body mass index (BMI) was 24.1 ± 3.1 kg/m^2^, and the mean of the physical activity scale was 4.0 ± 1.3 (Table 1).

**Table 1:** Demographic data on study participants.

Variable	n = 49
Age (years), M (SD)	37.4 (12.0)
Height (cm), M (SD)	169.9 (9.4)
Weight (kg), M (SD)	70.3* (13.9)
Physical Activity Level (1–6), M (SD)	4.0 (1.3)
BMI (kg/m^2^), M (SD)	24.1* (3.1)
Body fat US Navy (%), M (SD)	24.3 (8.9)
Dominant Leg (left – right)	1 – 48
Start Leg (left – right)	23 – 26
Calf circumference (cm), M (SD)	36.5 (3.0)
Tobacco use (smoker – snuff – no)	2 – 4 – 43
Gender (female – male)	21 – 28

### 3.2 Impedance assessed at different NMES currents applied

Impedance measurements using dry electrodes demonstrated a consistent decrease with increasing NMES current (Fig. 3A, Fig. 3BA–B). At 2.5 mA, mean impedance was 16.7 kΩ (95% CI: 14.0–19.3) on the right leg and 19.2 kΩ (95% CI: 16.6–21.8) on the left. At 17.5 mA, impedance decreased to 3.7 kΩ (95% CI: 1.9–5.5) on the right and 3.9 kΩ (95% CI: 2.7–5.0) on the left. Application of a gel pad to the left leg produced a nearly horizontal impedance curve: 5.8 kΩ (95% CI: 3.2–8.4) at 2.5 mA and 4.2 kΩ (95% CI: 2.7–5.6) at 17.5 mA (Fig. 1A, “Gel pad”). Exfoliating the skin under the dry electrode on the right leg resulted in a significantly steeper decline in impedance: from 10.2 kΩ (95% CI: 8.7–11.6) at 2.5 mA to 3.1 kΩ (95% CI: 2.0–4.3) at 17.5 mA (Fig. 1B, “Dry Exfoliation”). On the left leg, exfoliation followed by gel pad application showed no significant improvement over the gel pad alone, with impedance values of 4.6 kΩ (95% CI: 4.1–5.0) at 2.5 mA and 3.0 kΩ (95% CI: 2.1–4.0) at 17.5 mA. Conversely, on the right leg, applying the gel pad after exfoliation showed lower impedance at 2.5 mA (4.2 kΩ, 95% CI: 3.7–4.6), though differences disappeared at NMES levels ≥ 12.5 mA. Table 2A, Table 2B, Table 3 represents the distribution of current intensities required to elicit plantar flexion. No participant demonstrated plantar flexion below 5 mA or above 20 mA. The majority of participants reached plantar flexion between 10 and 15 mA, indicating an approximately normal distribution of activation thresholds.

**Fig. 3A:**
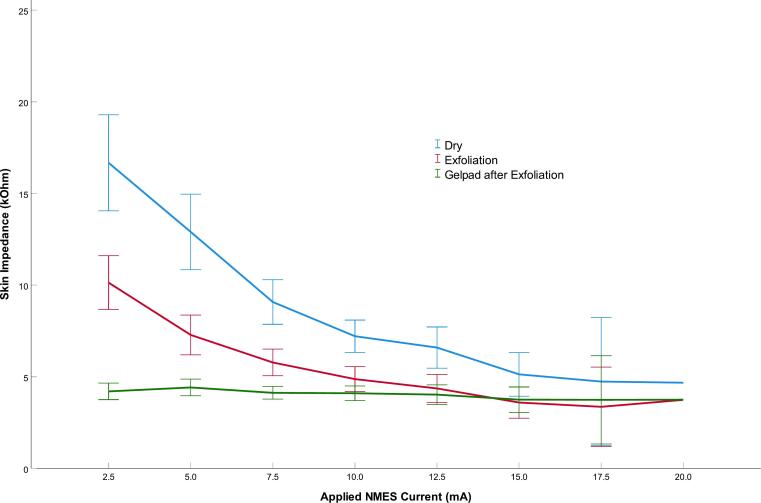
Distribution of impedances (kΩ) across applied NMES currents (mA) for all right-leg interventions. Corresponding muscle activation thresholds are detailed in Table 2A. Error bars indicate 95% CI.

**Table 2A:** Number of participants reaching plantar flexion between each incremental increase of current, left leg.

Current, mA	**<= 2.5**	**<= 5.0**	**<= 7.5**	**<= 10.0**	**<= 12.5**	**<= 15.0**	**<= 17.5**	**<= 20.0**
Dry	0	0	0	7	9	15^a^	11	6
Gelpad	0	0	1	7	10	18	6	7
Exfoliation after gelpad	0	0	2	6	14	15	5^a^	6

*Note.* N = 49 for each intervention. Note that no participant demonstrated plantar flexion at a lower intensity than 5 mA or higher than 20 mA.

a One subject aborting the test at specified intensity interval due to discomfort.

**Fig. 3B:**
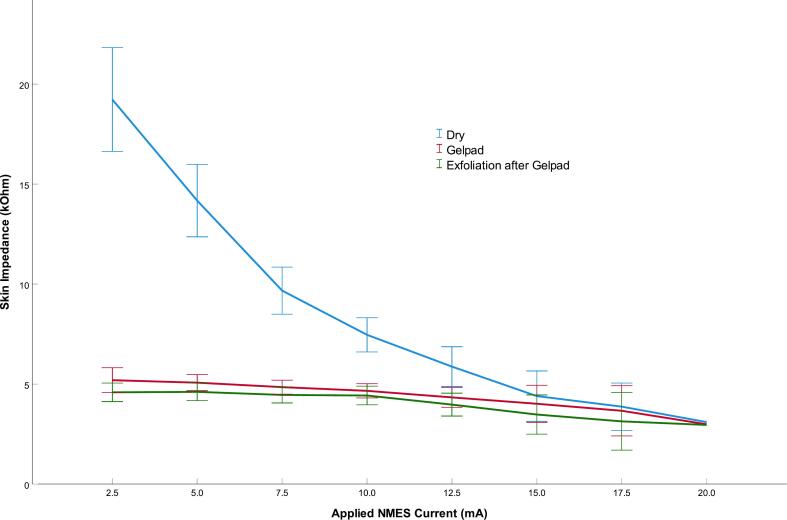
Distribution of impedances (kΩ) across applied NMES currents (mA) for all left-leg interventions. Corresponding muscle activation thresholds are detailed in Table 2B. Error bars indicate 95% CI.

**Table 2B:** Number of participants reaching plantar flexion between each incremental increase of current, right leg.

Current (mA)	**<= 2.5**	**<= 5.0**	**<= 7.5**	**<= 10.0**	**<= 12.5**	**<= 15.0**	**<= 17.5**	**<= 20.0**
Dry	0	0	0	1	13	17	10	8
Exfoliation	0	0	0	5	17	14	7	6
Gelpad after Exfoliation	0	0	0	6	15	16	8	4

*Note.* N = 49 for each intervention. Note that no participant demonstrated plantar flexion at a lower intensity than 7.5 mA or higher than 20 mA.

### 3.3 Impedance at plantar flexion (ZaPF)

**Fig. 4:**
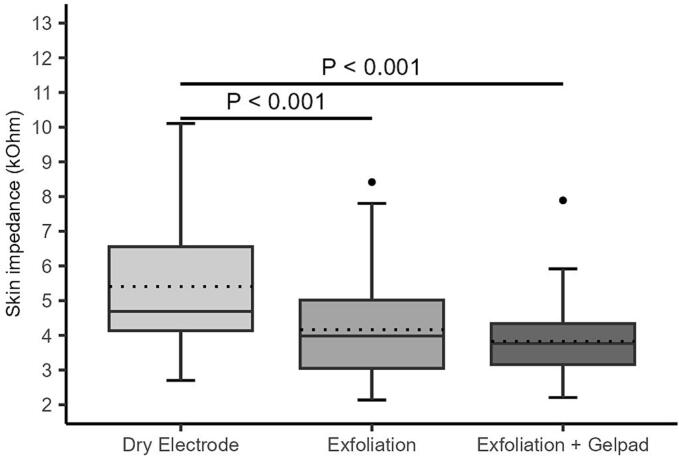

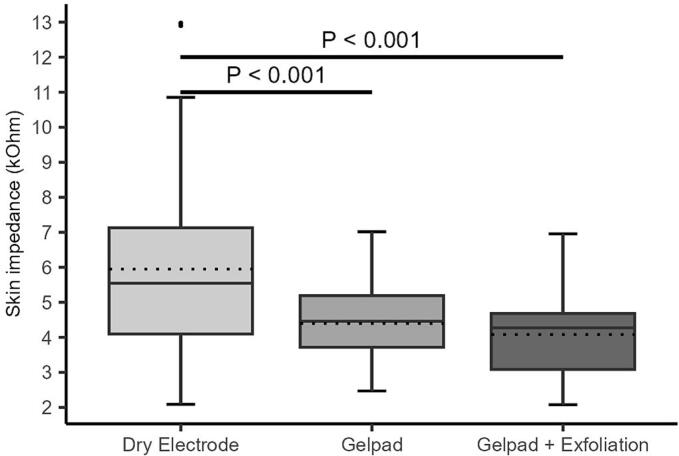
A-B. Distribution of impedance during plantar flexion across all interventions on the right (A) and left (B) legs. Boxplots display the median and interquartile range (IQR); dotted lines indicate mean impedance values. Individual dots represent outliers. Horizontal bars with corresponding p-values denote statistically significant differences between groups.

All interventions significantly reduced median impedance during plantar flexion (ZaPF) compared to dry electrodes (Fig. 4A–B). On the right leg, median ZaPF was reduced from 4.69 kΩ (IQR: 4.13–6.55) to 3.84 kΩ (IQR: 2.67–4.92) after exfoliation and to 3.75 kΩ (IQR: 3.03–4.32) after exfoliation plus gel pad (p < 0.001 for both; p = 0.253 between interventions). On the left leg, ZaPF decreased from 5.54 kΩ (IQR: 4.10–7.13) to 4.46 kΩ (IQR: 3.72–5.19) with the gel pad and to 4.27 kΩ (IQR: 3.09–4.68) with the combined intervention (p < 0.001 for both vs. baseline; p < 0.001 between interventions). Supplementary Fig. 1A-B displays individual ZaPF distributions.

### 3.4 NMES current required for plantar flexion (IaPF)

**Fig. 5:**
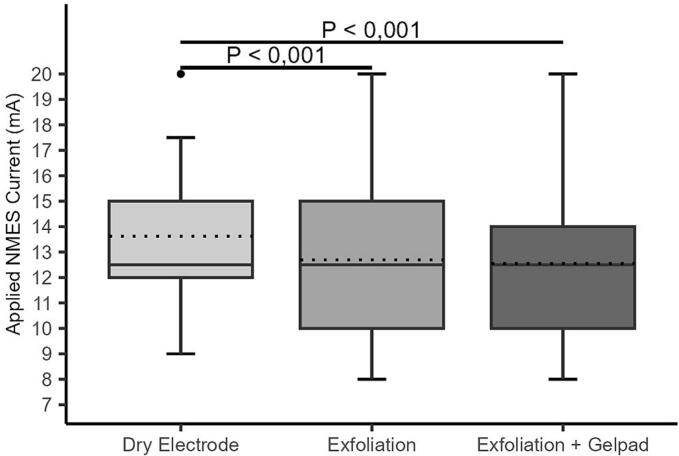

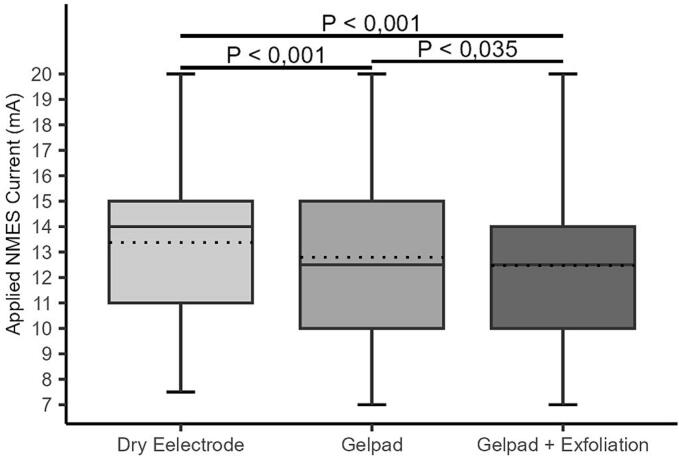
A–B. Distribution of applied neuromuscular electrical stimulation (NMES) current (mA) required to achieve plantar flexion across all interventions on the right (A) and left (B) legs. Boxplots display the median and interquartile range (IQR); dotted lines indicate mean current values. Individual dots represent outliers. Horizontal bars with corresponding *p*-values denote statistically significant differences between groups.

All interventions significantly lowered the current required to elicit plantar flexion (IaPF) compared to dry electrodes (p < 0.001) (Fig. 5A–B). On the right leg, median IaPF with dry electrodes was 12.5 mA (IQR: 12–15), remaining unchanged in value after exfoliation and exfoliation plus gel pad (both 12.5 mA, IQR: 10–15 and 10–14, respectively), although distributions shifted significantly lower (p < 0.001). No significant difference was observed between the two interventions (p = 0.152). On the left leg, IaPF decreased from 14.0 mA (IQR: 11–15) to 12.5 mA (IQR: 10–15) with the gel pad and to 12.5 mA (IQR: 10–14) with the combined intervention (p < 0.001 for both vs. baseline). A significant downward shift was detected between gel pad alone and combined (p = 0.035).

### 3.5 Numerical pain rating scale at plantar flexion (PaPF)

**Fig. 6:**
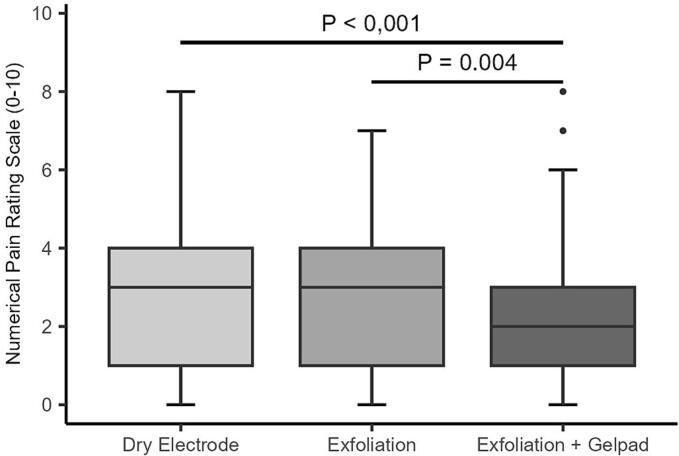

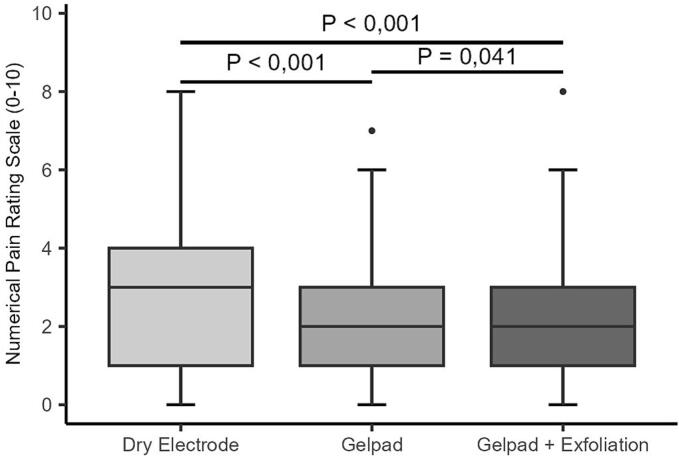
A–B. Distribution of reported PaPF across all interventions on the right (A) and left (B) legs. Boxplots display the median and interquartile range (IQR). Individual dots represent outliers. Horizontal bars with corresponding *p*-values denote statistically significant differences between groups.

All gel pad interventions significantly reduced pain ratings during plantar flexion (PaPF) (p < 0.001) (Fig. 6A–B). For the right leg, both the dry electrode and exfoliation yielded PaPF scores of 3 (IQR: 1–4), with no significant difference (p = 1.0). Exfoliation plus gel pad reduced median PaPF to 2 (IQR: 1–3) (p < 0.001 vs. exfoliation alone; p = 0.004 between interventions). On the left leg, PaPF fell from 3 (IQR: 1–4) to 2 (IQR: 1–3) in both gel pad and combined groups (p < 0.001). A subtle but significant upward shift in score distribution occurred after the combined intervention (p = 0.041), with 15 participants reporting higher PaPF. Notably, >20% of participants reported PaPF ≥ 4 even after gel pad use (Supplementary Fig. 2A-C and Fig. 3A-C).

### 3.6 Factors associated with impedance at plantar flexion

Multiple regression analysis was conducted to determine predictors of ZaPF. Variables included age, sex, BMI, U.S. Navy body fat percentage, Physical Activity Scale, applied NMES current, and starting leg. Higher physical activity levels consistently predicted lower ZaPF across all interventions and both legs (Table 3, Supplementary Table 1–2). Higher age and female sex were associated with increased impedance under certain conditions (Supplementary Table 1–2). Training status explained 34% of ZaPF variance with dry electrodes on the right leg. On the left leg, training status, starting leg, and NMES current explained 52.4% of the variance. Starting with the left leg reduced ZaPF by 1.54 kΩ. Each one-point increase in training score was linked to a decrease in ZaPF by 0.57 kΩ (right) and 0.59 kΩ (left).

**Table 3:** Multiple linear regression analyses with stepwise regression of impedance at plantar flexion across the dry electrode intervention on the right and left leg.

**Model /** Predictors	**R^2^ /** **Adj. R^2^**	**Model** **p-value**	**B (SE)**	**β (Coefficient)**	**95% CI**	**Predictor** **p-value**
**Impedance (kOhm)** *Dry electrode, right leg*	0.340 / 0.204	0.026*				
Sex a			0.308 (4.067)	0.080	[-2.22, 2.84]	0.807
Age (Years)			0.042 (0.027)	0.263	[-0.01, 0.10]	0.125
Weight (Kg)			−0.061 (0.064)	−0.440	[-0.19, 0.07]	0.348
Body Mass Index			0.251 (0.252)	0.402	[-0.26, 0.76]	0.325
US Navy Body Fat			−0.056 (0.059)	−0.251	[-0.18, 0.06]	0.353
Physical Activity Level			−0.573 (0.218)	−0.405	[-1.01, −0.13]	0.012*
Starting Leg ^b^			−0.307 (0.530)	−0.080	[-1.38, 0.77]	0.566
Applied NMES Current			−0.168 (0.099)	−0.242	[-0.37, 0.03]	0.097
**Impedance (kOhm)** *Dry electrode, left leg*	0.524 / 0.424	<0.001**				
Sex a			1.006 (1.429)	0.195	[-1.89, 3.90]	0.486
Age (Years)			0.057 (0.030)	0.266	[-0.00, 0.12]	0.067
Weight (Kg)			−0.065 (0.076)	−0.360	[-0.22, 0.09]	0.395
Body Mass Index			0.434 (0.295)	0.526	[-0.16, 1.03]	0.150
US Navy Body Fat			−0.134 (0.068)	−0.457	[-0.27, 0.00]	0.056
Physical Activity Level			−0.586 (0.249)	−0.312	[-1.09, −0.08]	0.024*
Starting Leg ^b^			−1.540 (0.631)	−0.301	[-2.82, −0.26]	0.019*
Applied NMES Current			−0.376 (0.101)	−0.463	[-0.58, −0.17]	0<.001**

*Note*. N = 49. Physical activity was coded on a 6-point scale, with higher values indicating higher activity levels.

a 1 = Male, 2 = Female. ^b^ 0 = Right, 1 = Left. * P < 0.05, ^**^ P < 0.001.

## 4 Discussion

This study systematically evaluated the effects of mechanical exfoliation, gel pad application, and their combination on skin impedance, required NMES current, and pain perception during NMES of the calf. Our findings demonstrate that both exfoliation and gel pad application significantly reduced skin–electrode impedance and the current intensity required to elicit plantar flexion. Notably, only the gel pad, used as a standalone intervention, resulted in a statistically significant reduction in perceived pain. These results support the use of hydrogel-based electrodes as a primary method for enhancing NMES efficiency and user comfort, particularly in contexts where tolerability is essential for compliance.

The main findings revealed that gel pads produced the largest impedance reduction (∼10 kΩ) in participants reaching PF at lower intensities (PF range: 5–20 mA), whereas those with PF at higher intensities gained little or no impedance reduction. During plantar flexion, all interventions reduced impedance with a mean of ∼ 1.5 kΩ and current required (∼1 mA) compared to the dry electrode condition. However, significant pain reduction (by ∼ 1 NPRS point) was observed only with gel pad use, either alone or combined with exfoliation. This highlights the clinical relevance of hydrating interfaces not only in improving electrical performance but also in modulating sensory perception.

Comparing the methodology of this study and the prior work conducted by Bîrlea et al (Bîrlea et al., 2014), applying the same circuit model, measuring the peak voltage reflects the combined influence of the stratum corneum resistance, the stratum corneum capacitance and the series resistance of the underlying tissue at a given moment. By ensuring that the electrodes stay in the exact same position across interventions, the changes in impedance found across all interventions are attributable to the change in the stratum corneum and the interface between the electrode and the stratum corneum.

The results of this study are consistent with prior studies indicating that hydration at the skin-electrode interface and possible sudomotor activation over the duration of stimulation reduces the capacitive impedance of the stratum corneum and enhances current transmission (Björklund et al., 2013, Euler et al., 2022, Sundström et al., 2023). Gel pads in particular, maintained low impedance values (∼5 kΩ) across all tested stimulation intensities, suggesting a stable interface and reduced variability in electrical output.

Interestingly, the combination of exfoliation and gel pad use further reduced the current needed for muscle activation compared to gel pads alone, though this did not lead to additional reductions in impedance or perceived pain. This systematic dissociation between impedance reduction and pain reduction, particularly the observation that mechanical exfoliation significantly decreased impedance without improving pain ratings, challenges a simple linear relationship between skin conductivity and perceived discomfort and suggests that additional cutaneous and central mechanisms shape NMES tolerability. These findings imply that exfoliation may offer limited benefits once hydration is optimized, or that the removal of the stratum corneum provides diminishing returns in the presence of a conductive medium, although combined strategies may still be useful in individuals with high baseline impedance. However, this conclusion requires further investigation in larger and more diverse clinical populations.

Impedance analysis showed distinct response patterns across conditions. In dry and exfoliation-only conditions, impedance decreased with increasing stimulation, consistent with non-linear resistive behavior of skin tissue (Bîrlea et al., 2014). Conversely, impedance remained low and stable with gel pad use, indicating less variability and more predictable delivery of stimulation. These results support the idea that hydration, rather than mechanical skin modification, is more effective for optimizing the electrode–skin interface.

The convergence of impedance values at higher NMES currents across all interventions suggests that impedance-lowering strategies are most effective at lower intensities. This finding has implications for clinical populations where minimizing stimulation thresholds and discomfort is critical, such as in chronic pain or pediatric settings.

Exploratory regression analyses identified higher physical activity levels as a consistent predictor of lower impedance across all models on both legs. This may be explained by prior work conducted by Schriwer et al., who demonstrated that higher physical activity levels are associated with a greater number of motor points in the calf muscle and stimulation at motor points results in lower impedance (Flodin et al., 2025). This finding suggests a clinically relevant implication: that individuals with low training status—those most likely to benefit from NMES—also present with higher impedance and potentially greater discomfort.

Higher applied NMES current were also associated with lower impedance in some models. However, these associations were of moderate magnitude, explained only a limited proportion of the variance, and were not uniform across interventions or legs. Age, sex, and body-composition variables showed, at most, borderline or condition-specific associations with impedance, potentially reflecting physiological factors such as skin thickness, hydration, or vascularity. Future studies should include dermatological profiling to enable personalized NMES protocols.

A major strength of this study was the controlled environment, with standardized temperature, humidity, electrode placement, and stimulation settings. The use of a well-defined cohort and multivariate statistical modeling enhances both internal validity and translational potential. However, limitations include the absence of direct measurements of skin hydration, oil content, and stratum corneum thickness, as well as direct measurement of skin impedance. Sweat gland activity and inter-individual pain perception variability were also not accounted for.

In conclusion, hydration of the electrode–skin interface is the most effective strategy for reducing impedance and improving user experience in NMES. Gel pads outperform dry electrodes significantly, and while exfoliation provides some added benefit, its utility may be limited when hydration is sufficient. Lower impedance is consistently linked to reduced stimulation thresholds and pain, underscoring the clinical and engineering need for more advanced skin-electrode interface solutions.

## CRediT authorship contribution statement

**Philip Wackendal:** Methodology, Data curation, Formal analysis, Investigation, Writing – original draft, Writing – original draft. **Ines Shopi:** Data curation, Investigation, Writing – original draft, Writing – original draft. **Robin Juthberg:** Conceptualization, Methodology, Data curation, Investigation, Writing – original draft. **Paul W Ackermann:** Conceptualization, Methodology, Writing – original draft, Writing – original draft, Supervision.

## Funding

Financial support from the Swedish Research Council (2017-00202), the regional agreement on medical training and clinical research, Stockholm (ALF Medicin 2022; FoUI-960998) and Hälsa och medicinteknik (HMT), Sweden (FoUI-979040) all to PA.

## Declaration of competing interest

The authors declare the following financial interests/personal relationships which may be considered as potential competing interests: PA and RJ declare a potential conflict of interest. They have been granted a patent related to neuromuscular electrical stimulation. UKPatent, publication number GB2601757. A system comprising of a controller and an electrical stimulation system. The remaining authors declare that the research was conducted in the absence of any commercial or ﬁnancial relationships that could be construed as a potential conflict of interest.
