# Supplementary material for: Interventions to mitigate pain and reduce skin impedance during neuromuscular electrical stimulation
Source: Clin Neurophysiol Pract. 2026 Feb 13;11:199–207. doi: 10.1016/j.cnp.2026.02.005 (PMC13053858; doi:10.1016/j.cnp.2026.02.005)
Supplement: Supplementary Data 1 [file mmc1.pptx]

## Slide 1
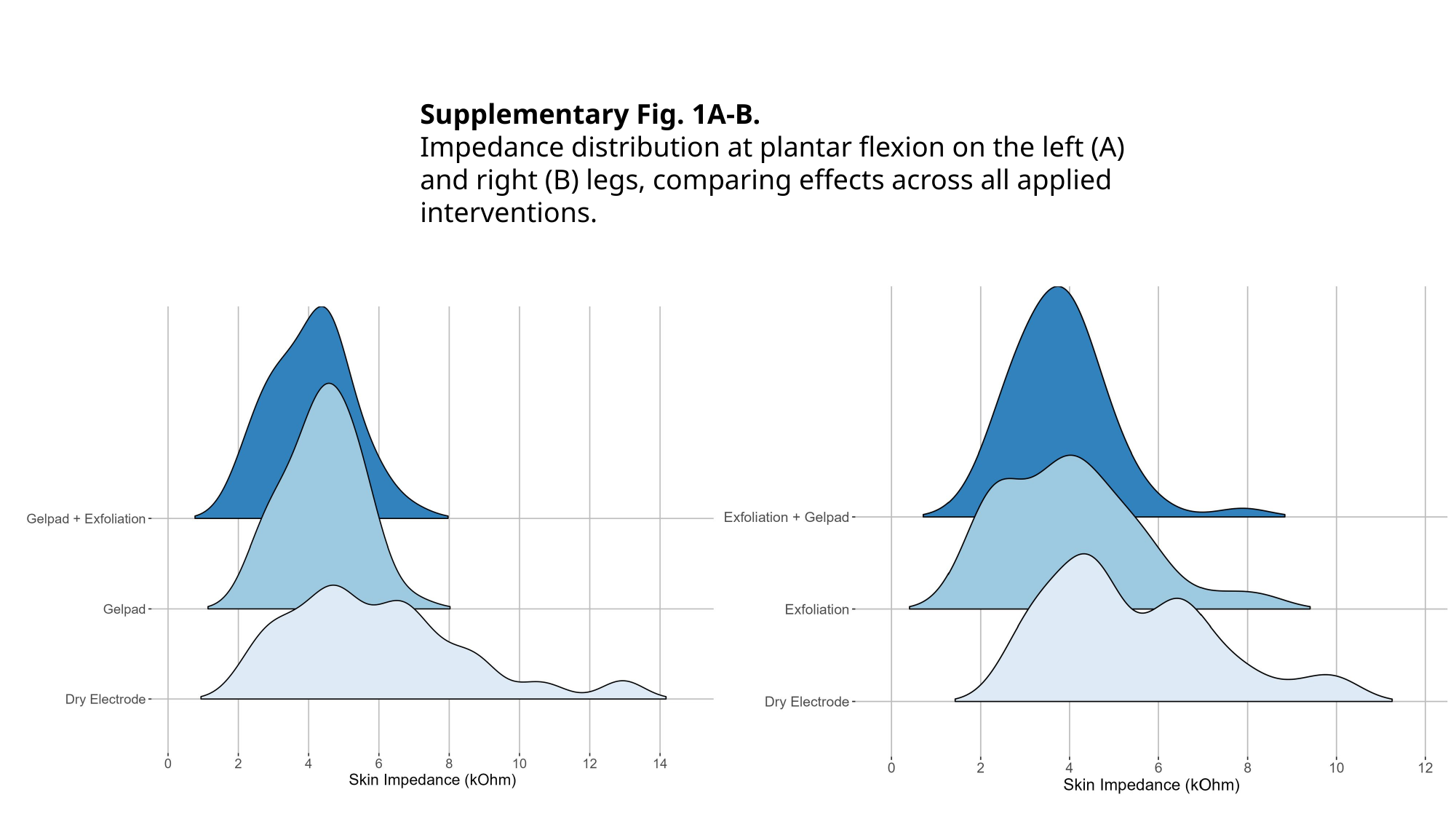

Supplementary Fig. 1A-B.
Impedance distribution at plantar flexion on the left (A) and right (B) legs, comparing effects across all applied interventions.

## Slide 2
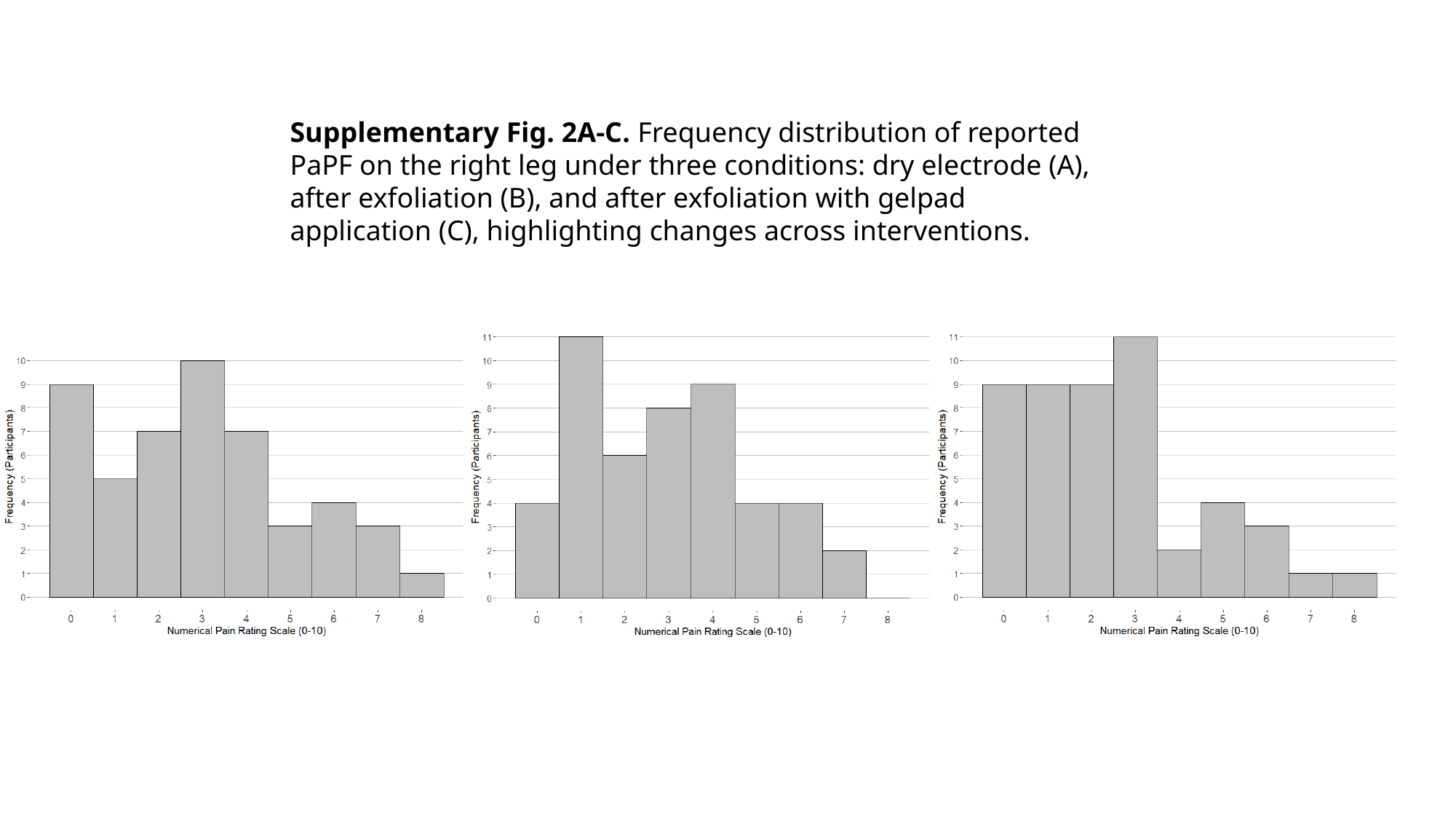

Supplementary Fig. 2A-C. Frequency distribution of reported PaPF on the right leg under three conditions: dry electrode (A), after exfoliation (B), and after exfoliation with gelpad application (C), highlighting changes across interventions.

## Slide 3
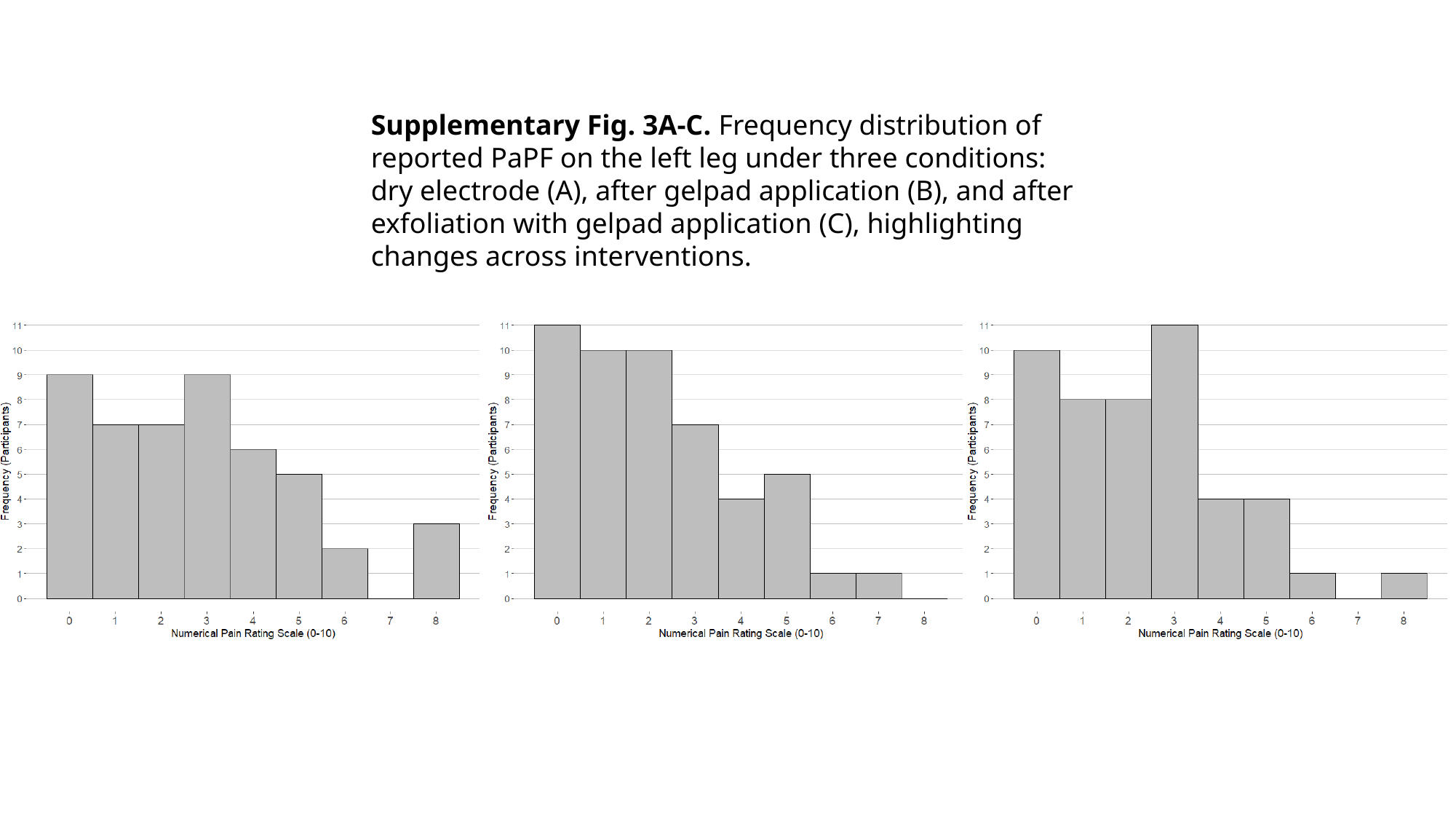

Supplementary Fig. 3A-C. Frequency distribution of reported PaPF on the left leg under three conditions: dry electrode (A), after gelpad application (B), and after exfoliation with gelpad application (C), highlighting changes across interventions.

## Slide 4
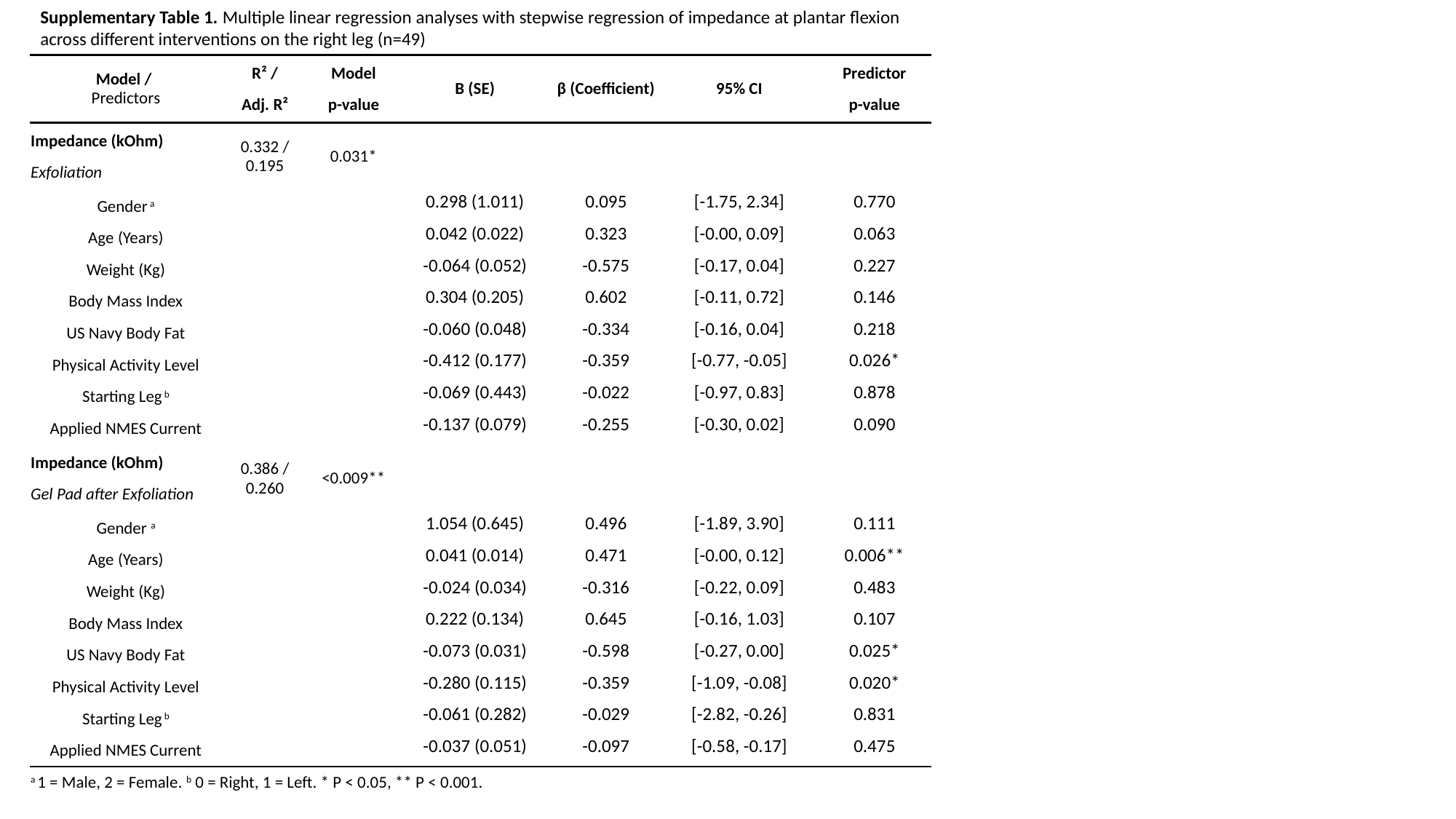

Supplementary Table 1. Multiple linear regression analyses with stepwise regression of impedance at plantar flexion across different interventions on the right leg (n=49)
| Model / Predictors | R² / Adj. R² | Model p-value | B (SE) | β (Coefficient) | 95% CI | Predictor p-value |
| --- | --- | --- | --- | --- | --- | --- |
| Impedance (kOhm) Exfoliation | 0.332 / 0.195 | 0.031\* | | | | |
| Gender a | | | 0.298 (1.011) | 0.095 | [-1.75, 2.34] | 0.770 |
| Age (Years) | | | 0.042 (0.022) | 0.323 | [-0.00, 0.09] | 0.063 |
| Weight (Kg) | | | -0.064 (0.052) | -0.575 | [-0.17, 0.04] | 0.227 |
| Body Mass Index | | | 0.304 (0.205) | 0.602 | [-0.11, 0.72] | 0.146 |
| US Navy Body Fat | | | -0.060 (0.048) | -0.334 | [-0.16, 0.04] | 0.218 |
| Physical Activity Level | | | -0.412 (0.177) | -0.359 | [-0.77, -0.05] | 0.026\* |
| Starting Leg b | | | -0.069 (0.443) | -0.022 | [-0.97, 0.83] | 0.878 |
| Applied NMES Current | | | -0.137 (0.079) | -0.255 | [-0.30, 0.02] | 0.090 |
| Impedance (kOhm) Gel Pad after Exfoliation | 0.386 / 0.260 | <0.009\*\* | | | | |
| Gender a | | | 1.054 (0.645) | 0.496 | [-1.89, 3.90] | 0.111 |
| Age (Years) | | | 0.041 (0.014) | 0.471 | [-0.00, 0.12] | 0.006\*\* |
| Weight (Kg) | | | -0.024 (0.034) | -0.316 | [-0.22, 0.09] | 0.483 |
| Body Mass Index | | | 0.222 (0.134) | 0.645 | [-0.16, 1.03] | 0.107 |
| US Navy Body Fat | | | -0.073 (0.031) | -0.598 | [-0.27, 0.00] | 0.025\* |
| Physical Activity Level | | | -0.280 (0.115) | -0.359 | [-1.09, -0.08] | 0.020\* |
| Starting Leg b | | | -0.061 (0.282) | -0.029 | [-2.82, -0.26] | 0.831 |
| Applied NMES Current | | | -0.037 (0.051) | -0.097 | [-0.58, -0.17] | 0.475 |
| a 1 = Male, 2 = Female. b 0 = Right, 1 = Left. \* P < 0.05, \*\* P < 0.001. | | | | | | |

## Slide 5
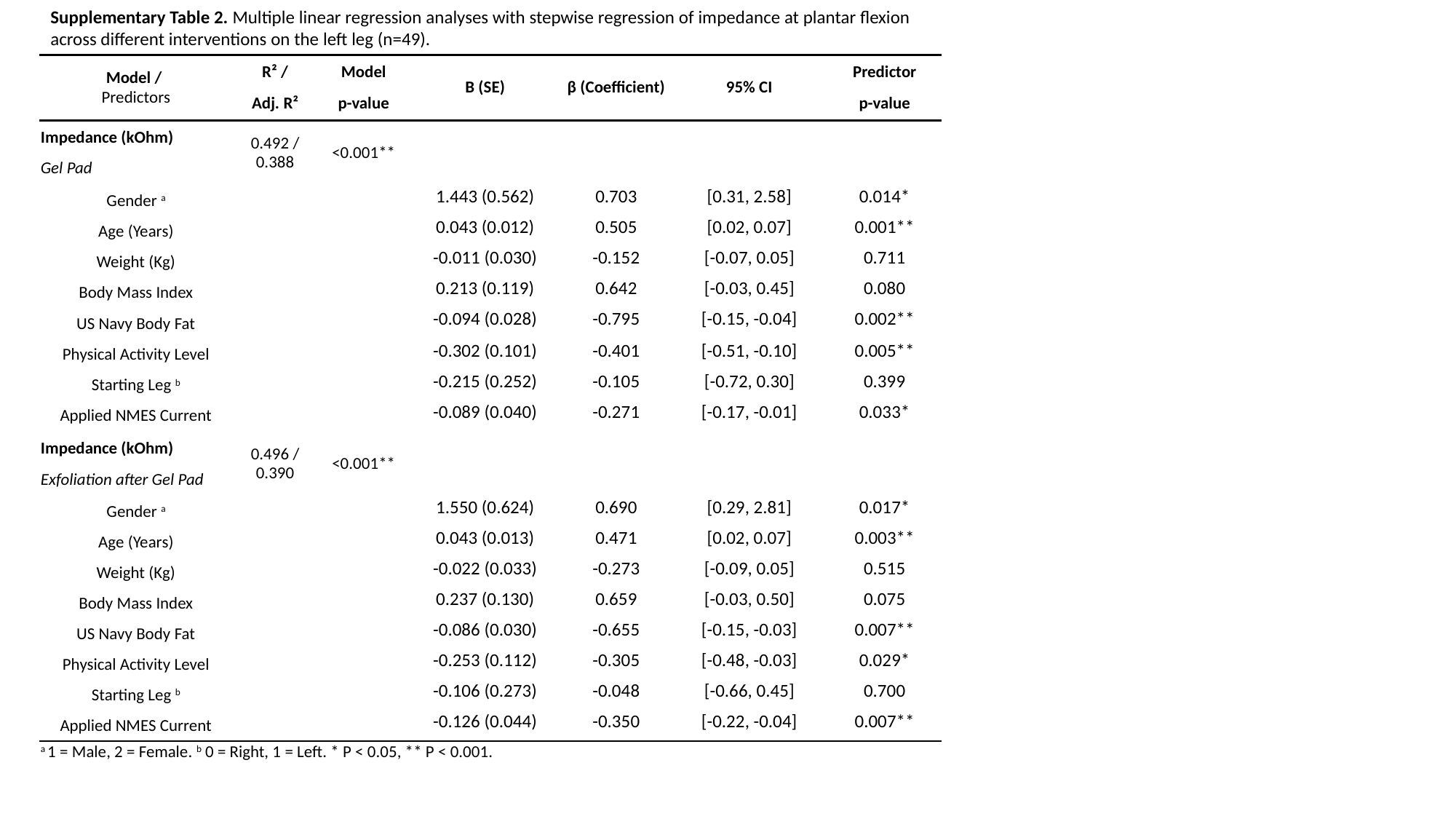

Supplementary Table 2. Multiple linear regression analyses with stepwise regression of impedance at plantar flexion across different interventions on the left leg (n=49).
| Model / Predictors | R² / Adj. R² | Model p-value | B (SE) | β (Coefficient) | 95% CI | Predictor p-value |
| --- | --- | --- | --- | --- | --- | --- |
| Impedance (kOhm) Gel Pad | 0.492 / 0.388 | <0.001\*\* | | | | |
| Gender a | | | 1.443 (0.562) | 0.703 | [0.31, 2.58] | 0.014\* |
| Age (Years) | | | 0.043 (0.012) | 0.505 | [0.02, 0.07] | 0.001\*\* |
| Weight (Kg) | | | -0.011 (0.030) | -0.152 | [-0.07, 0.05] | 0.711 |
| Body Mass Index | | | 0.213 (0.119) | 0.642 | [-0.03, 0.45] | 0.080 |
| US Navy Body Fat | | | -0.094 (0.028) | -0.795 | [-0.15, -0.04] | 0.002\*\* |
| Physical Activity Level | | | -0.302 (0.101) | -0.401 | [-0.51, -0.10] | 0.005\*\* |
| Starting Leg b | | | -0.215 (0.252) | -0.105 | [-0.72, 0.30] | 0.399 |
| Applied NMES Current | | | -0.089 (0.040) | -0.271 | [-0.17, -0.01] | 0.033\* |
| Impedance (kOhm) Exfoliation after Gel Pad | 0.496 / 0.390 | <0.001\*\* | | | | |
| Gender a | | | 1.550 (0.624) | 0.690 | [0.29, 2.81] | 0.017\* |
| Age (Years) | | | 0.043 (0.013) | 0.471 | [0.02, 0.07] | 0.003\*\* |
| Weight (Kg) | | | -0.022 (0.033) | -0.273 | [-0.09, 0.05] | 0.515 |
| Body Mass Index | | | 0.237 (0.130) | 0.659 | [-0.03, 0.50] | 0.075 |
| US Navy Body Fat | | | -0.086 (0.030) | -0.655 | [-0.15, -0.03] | 0.007\*\* |
| Physical Activity Level | | | -0.253 (0.112) | -0.305 | [-0.48, -0.03] | 0.029\* |
| Starting Leg b | | | -0.106 (0.273) | -0.048 | [-0.66, 0.45] | 0.700 |
| Applied NMES Current | | | -0.126 (0.044) | -0.350 | [-0.22, -0.04] | 0.007\*\* |
| a 1 = Male, 2 = Female. b 0 = Right, 1 = Left. \* P < 0.05, \*\* P < 0.001. | | | | | | |
